# Impact of Cluster Farming on Wheat Productivity and Welfare Among Smallholder Farmers in Ethiopia

**DOI:** 10.1155/tswj/8897802

**Published:** 2025-12-10

**Authors:** Mesele Belay Zegeye, Mahlet Getahun Deredera, Anteneh Bizualem Asefa, Abate Belaye Tefera

**Affiliations:** ^1^ Department of Economics, Woldia University, Woldia, Ethiopia, wldu.edu.et; ^2^ Department of Economics, Debre Berhan University, Debre Berhan, Ethiopia, dbu.edu.et

**Keywords:** adoption, cluster farming, endogenous switching regression, productivity, smallholder wheat farmers, welfare

## Abstract

This study was conducted with the objective of investigating the impact of adopting cluster farming on wheat productivity and the welfare of smallholder households in the North Shewa zone of the Amhara region in Ethiopia. The study used primary data collected from 394 households sampled using a multistage sampling technique. The data was collected using structured questionnaires and key informant interviews. In order to investigate the impact of adopting cluster farming on households′ wheat productivity measured by their production per hectare and welfare indicated by their food consumption expenditure, the study has used an endogenous switching regression (ESR) model. The result of the analysis indicates that factors such as the household head′s age, education level, perception of cluster farming, household size, farming experience, farm size allocated to wheat production, participation in farmers′ unions, access to irrigation and information about cluster farming, soil quality, training, and proximity to resources have a significant influence on farm households′ decision to adopt cluster farming. The results confirm that the adoption of cluster farming significantly increases wheat yields and directly enhances household per capita food consumption expenditure compared to nonadopters. Furthermore, the findings suggest that nonadopters could have achieved higher wheat productivity and improved food consumption levels had they adopted the practice. Based on these results, the study recommends that the government and relevant stakeholders collaborate with rural farming households to promote cluster farming in the study area, thereby improving smallholder farmers′ wheat productivity and overall welfare.

## 1. Introduction

Agriculture is the backbone of Ethiopia′s economy, serving as the primary source of food, employment, and export earnings [1, 2]. According to the Ministry of Agriculture [3], crop production dominates the agricultural sector, contributing approximately 60% of total annual output. However, this sector is largely driven by smallholder farmers who rely on rain‐fed agriculture, highly vulnerable to climate change, poor adoption of modern inputs/technologies, and traditional practices, resulting in low productivity and persistent food insecurity in the country [4, 5].

To address these structural challenges, the Ethiopian government, in collaboration with development partners, has initiated and implemented several agricultural transformation efforts. A key initiative is the Agricultural Commercialization Cluster (ACC) program, launched by the Agricultural Transformation Agency (ATA) in 2021/2022. This program is part of a broader reform agenda targeting input systems, irrigation, mechanization, soil fertility, extension services, and market infrastructure [6, 7].

The ACC program adopts a geographically targeted, value chain‐based approach to modernize smallholder agriculture. At its core is the concept of cluster farming, a model that encourages farmers in close proximity to coordinate crop production by adopting uniform agronomic practices [8]. It enables them to pool resources, land, labor, inputs, and knowledge, thereby reducing costs and improving efficiency [7]. It also strengthens farmers′ bargaining power, improving access to markets, credit, and government support [9–12]. By jointly investing in infrastructure like storage and transport, farmers can add value and expand market reach [13]. It focuses on high‐priority value chain crops, such as wheat, maize, and teff, and provides integrated support services including modern technologies or inputs, extension services, mechanization, and market linkages. As noted by ATA [8], this model is aimed at enhancing productivity, boosting commercialization, improving food security, and contributing to poverty reduction.

Cluster farming is often confused with other models such as cooperative farming and land consolidation. Under the former Ethiopian socialist regime, cooperative farming involved mandatory joint ownership and collective land management, which resulted in inefficiency and resistance [14]. In contrast, cluster farming is based on voluntary participation and preserves individual land ownership while encouraging coordinated production [8, 13]. Similarly, land consolidation involves physically merging fragmented plots into larger holdings under single management [15], whereas cluster farming allows individual farming while synchronizing inputs and production timelines to improve efficiency.

The benefits of cluster farming extend beyond production efficiency. By pooling resources such as land, labor, and information, farmers can lower production costs, adopt improved technologies, and access shared infrastructure like storage and transportation facilities. Additionally, collective action enables better market access, stronger bargaining power, and improved access to financial services such as credit [9–13]. These advantages make cluster farming a promising strategy for enhancing resilience, improving food security, and reducing rural poverty.

Wheat is Ethiopia′s second most important source of calories, contributing approximately 14% of the nation′s total dietary intake [16, 17]. The country is the largest wheat producer in sub‐Saharan Africa [18], with an estimated 5.5 million tons produced annually [19]. The ACC initiative has been piloted in over 211 woredas across four major regions: Amhara, Oromia, Southern Nations, Nationalities and Peoples Region (SNNPR), and Tigray, primarily for wheat production under the cluster farming model. It follows a monitoring and learning approach to scale implementation while adapting interventions to local agroecological conditions and market opportunities. Within this framework, cluster farming in the Amhara region has specifically prioritized wheat production, recognizing its critical role in ensuring national food security, meeting growing domestic demand, and enhancing rural livelihoods [8]. This effort aligns with the country′s government′s recent emphasis on achieving wheat self‐sufficiency and reducing dependence on imports [20].

In line with these national efforts, the Amhara region, particularly the North Shewa Zone, has emerged as a key pilot area for cluster farming due to its potential in wheat production. Wheat is considered a strategic crop for ensuring food self‐sufficiency and reducing Ethiopia′s reliance on imports [20]. Despite this, Ethiopia continues to face a production deficit, necessitating large‐scale wheat imports each year [18, 21]. In North Shewa, wheat productivity remains low due to limited adoption of modern technologies and continued reliance on traditional farming methods [22, 23]. Even with its potential, the implementation of cluster farming in the country remains limited in scale and informally structured, with weak market linkages [7, 24]. To scale the practice sustainably, it is essential to understand the factors driving its adoption, assess its broader impacts, and identify challenges faced by participants.

This study is aimed at contributing to the growing body of empirical literature on cluster farming in several key ways. First, it presents the first systematic analysis of the factors influencing the adoption of cluster farming and its impact on the productivity and welfare of smallholder farmers in the North Shewa Zone of the Amhara Region, one of the key pilot areas for the cluster farming initiative in Ethiopia. Given that participation in cluster farming is voluntary and adoption decisions are made at the household level, with the government mainly providing support services rather than enforcing participation, the study employs a household‐level analysis.

Second, while previous studies (e.g., [25–29]) have examined the determinants and impacts of cluster farming on productivity, welfare, and commercialization using methods such as propensity score matching (PSM), inverse probability‐weighted regression adjustment (IPWRA), ordinary least squares (OLS), and descriptive statistics. These studies mostly relied on before‐and‐after comparisons. As a result, they often failed to adequately control for both observable and unobservable heterogeneities that may influence the decision to adopt and its subsequent outcomes. These models are limited by self‐selection bias, endogeneity, and the lack of reliable counterfactuals. In contrast, this study employs an endogenous switching regression (ESR) model, which addresses selection bias and endogeneity issues more effectively. It also enables the estimation of actual versus counterfactual outcomes, offering more accurate insights into the true impact of cluster farming. This is especially relevant for agricultural development policy, as it helps to justify the scaling and expansion of cluster farming to other regions and crop types [22, 23].

Third, this study contributes to the existing literature on how cluster farming can significantly improve smallholders′ productivity, welfare, food security, nutritional outcomes, and consumption levels (e.g., [25, 26, 30–33]). It adds cluster farming as a promising policy tool to facilitate smallholders′ transition into large‐scale production and enhance rural livelihoods in developing countries like Ethiopia. Accordingly, the objective of this study is to investigate the factors influencing the adoption of cluster farming among smallholder farmers in North Shewa Zone, Amhara region, and to assess its impact on wheat productivity, measured by output per hectare, and household welfare, indicated by monthly per capita consumption expenditure, using the ESR approach.

The rest of the paper is organized as follows: Section 2 presents a review of relevant empirical literature. Section 3 outlines the methodology, including the study area, sampling procedure, and econometric framework. Section 4 presents the results and discussion. Section 5 concludes the paper with key policy implications.

## 2. Literature Review

Low agricultural productivity among smallholder farmers of Ethiopia has promoted the implementation of cluster farming through initiatives like ACC to enhance output and improve households′ welfare. Although recent empirical studies have begun to investigate cluster farming′s adoption and impact, little is known about its effects on smallholder farmers′ productivity and welfare, and its potential in the North Shewa Zone of the Amhara region, one of the key pilot areas for ACC, remains unexplored. This study addresses this gap by evaluating the impact of cluster farming on wheat production and household welfare in this area using ESR, a robust approach to account for selection bias and unobserved heterogeneity. The following literature review synthesizes existing empirical studies on cluster farming in Ethiopia, exploring factors driving adoption, productivity gains, commercialization, welfare improvements, and challenges, while identifying gaps this study aims to fill.

Cluster farming has shown significant potential in enhancing agricultural productivity across various regions and crop items, as evidenced by multiple studies. Cheffo et al. [34] found that cluster farming significantly increased malt and barley production in the Arsi and West Arsi zones of the Oromiya region in Ethiopia, with members of cluster farming achieving higher yields than nonadopters. Louhichi et al. [26] highlighted that cluster farming can increase production size by 1.8%–25%, depending on crop type and adoption scenario. The variation reflects differences in land productivity gains from partial versus full adoption and crop responsiveness. Wogderes [29] reported higher wheat yields among cluster farmers in the Siyadebr ena Wayu district of the Amhara region, and Warkineh et al. [28] noted improvement in chickpea quality and quantity in the North Gonder Zone. Degefu et al. [24] identify key adoption determinants of cluster farming in the Arsi zone, such as education, cooperative membership, and access to training, while noting that market distance negatively affects participation rates. Their analysis also revealed that cluster farming significantly improves wheat productivity with adopters potentially facing a 40% productivity drop without it and nonadopters could have increased productivity by 46% and net benefit by 102% through adoption. Collectively, these findings underscore cluster farming′s role in improving productivity.

These boosts in productivity achieved through cluster farming are found to play a pivotal role in improving crop commercialization and market integration. Empirical studies highlight this potential of cluster farming to shift smallholder farmers from subsistence to market‐oriented production. Dureti et al. [35] analyzed cluster farming on key crops like teff, wheat, maize, barley, and sesame across multiple regions of the country. The results of their analysis indicate that the adoption of the practice has a positive and significant impact on households′ commercialization index, market surplus value, and market price, indices used to represent household‐level crop commercialization. Similarly, Endalew et al. [7] demonstrated its positive effect on teff commercialization in priority cluster farming regions. Abate [25] also indicates the positive and significant role cluster farming has in improving maize commercialization. These outcomes can be related to improved production associated with cluster farming as stated in the references above, specialization related to cluster farming [36], and reduced postharvest loss.

While cluster farming enhances commercialization and market engagement, its impact on household welfare and poverty reduction is also equally significant. Empirical studies have shown that cluster farming improves welfare by increasing income, food security, and asset accumulation. For instance, Louhichi et al. [26] found that the improvement in production and income can help to enhance households′ food consumption and nutrition indicators and reduce the poverty gap by up to 2.1%. Similarly, Gidelew et al. [37] showed that it significantly improves multiple dimensions of household‐level food security like quantity, acceptability, and stability. Ali and Tefera [38] further highlight its positive effect on food security, with adopters scoring significantly higher dietary diversification scores than nonadopters. Expanding the scope, Jr Tabe‐Ojong and Dureti [35] used nationwide data and an instrumental variable approach to confirm that cluster farming can reduce poverty and the poverty gap in Ethiopia. Additionally, Tefera et al. [39], by applying PSM, revealed that cluster farming has a positive role in building household assets.

While the welfare gains enhance farmers′ economic capacity, the role of cluster farming in promoting mechanization and technology uptake is also critical to further improve agricultural outcomes. Cluster farming in Ethiopia has significantly enhanced agricultural mechanization, a critical factor in boosting productivity and reducing postharvest losses for smallholder farmers [40]. These gains stem from resource pooling which enables farmers to share costs and access modern equipment in regions traditionally marked by low mechanization rates.

Cluster farming in Ethiopia faces significant challenges that constrain its adoption and effectiveness limiting its potential to enhance agricultural outcomes for smallholder farmers. These include difficulty in coordination among farmers with diverse resource endowments and levels of commitment [24], limited market access and infrastructure [7], and inconsistent provision of inputs and technical support [38]. A study by Cheffo et al. [34] has also noted unequal distribution of benefits within clusters, which often favor wealthier farmers. Issues related to land fragmentation and vulnerability to climate shocks have also been cited as barriers [37]. Considering these challenges is essential for designing context‐specific cluster farming models and scaling it as a strategy.

While the existing empirical studies discussed earlier provide valuable insight into cluster farming′s impact, their reliance on conventional analytical approaches like PSM often fails to adequately address selection bias arising from both observed and unobserved heterogeneity. Consequently, their estimates may not fully capture the true effect of cluster farming or provide accurate counter factual comparisons. To address this methodological limitation, this study employs the ESR model, which explicitly accounts for selection bias and allows for more robust impact estimates. Previous studies like Endalew et al. [7] used the method to investigate the impact of cluster farming on teff production, and this study extends its use to evaluate the impact of cluster farming on wheat production and household consumption expenditure. By focusing on North Shewa, a key ACC pilot zone, the analysis in this study delivers context‐specific evidence to guide targeted scaling of cluster farming in Ethiopia. The evidence will enable policymakers to identify the causal impact of cluster farming, identify its potential adopters who could benefit the most, and design spatially tailored interventions to improve program effectiveness.

### 2.1. Conceptual Framework

**Figure 1:**
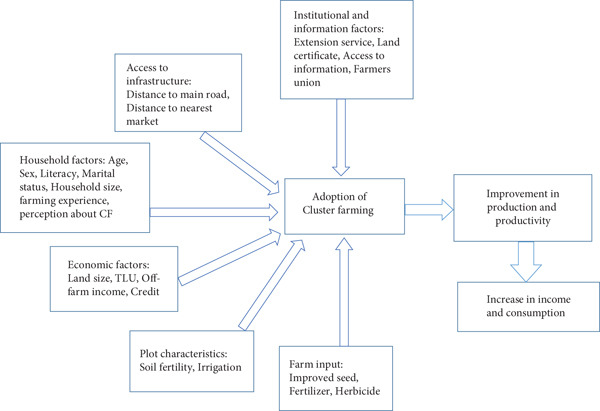
Conceptual framework of the study, adapted from Zegeye et al. [41].

Based on the literature review, cluster farming can enhance agricultural productivity, commercialization, and household welfare among smallholder farmers. Adoption is influenced by general factors, including household‐specific characteristics, plot attributes, institutional and informational support, infrastructure, economic conditions, and farm input availability. While cluster farming improves crop yields, income, and resource‐use efficiency, challenges such as coordination issues, limited market access, and unequal benefit distribution may constrain its effectiveness. Figure 1 illustrates these factors and their expected impact on productivity and welfare.

## 3. Methodology

### 3.1. Description of the Study Area

**Figure 2:**
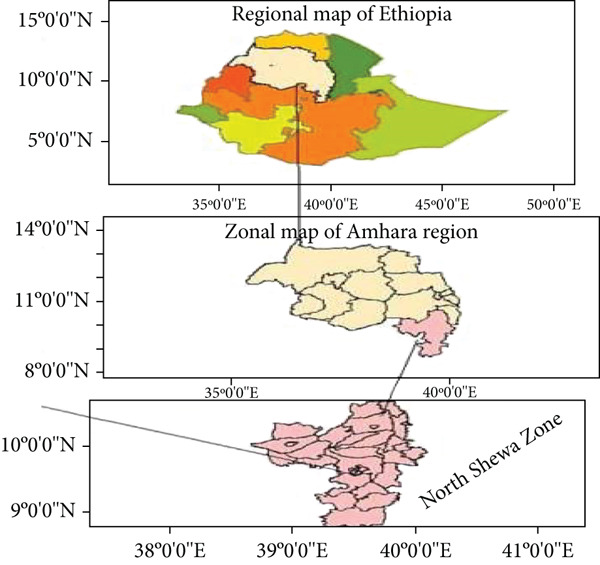
Geographical location of the study area.

This study was conducted using data from sampled rural households in North Shewa Zone, located in the Amhara region of Ethiopia. Geographically, the zone is bordered by the Oromiya region to the south and west, South Wollo Zone to the north, the Oromiya Special Zone to the north east, and the Afar region to the east, presented in Figure 2. The area experiences average annual rainfall ranging between 400 and 700 mm, with temperature fluctuating from −8°C to 35.7°C throughout the year [42]. Approximately 88% of the population resides in the rural area with agriculture as their principal source of livelihood. According to NSZCO [42], the zone′s major agricultural products include wheat, barley, teff, sorghum, maize, beans, peas, chickpeas, and a variety of vegetables. North Shewa is also recognized as one of Ethiopia′s key wheat‐producing zones. In 2021, the average wheat yield of the area was 3060 kg/ha, surpassing the regional average of 2830 kg/ha reported during the same period [43].

The study area was purposively selected based on the presence of cluster farming pilot programs. North Shewa was one of the zones identified by the ATA to pilot and scale up the cluster farming model, aiming to enhance agricultural productivity and promote the adoption of improved technologies among smallholder farmers. Since its inception in 2021, the cluster farming initiative has covered over 2800 ha of land nationwide and has promoted cluster wheat production by improving farmers′ access to agricultural inputs, technologies, and extension services while fostering peer learning and knowledge exchange [44].

### 3.2. Sampling and Data Collection Method

The study has employed a three‐stage sampling technique to select representative farming households from the study area. In the first stage, four woredas (districts), Siyadebirna Wayu (with 15,412 wheat farming households), Moret ena Jiru (with 29,260 wheat farming households), Mida Weremo (with 31,567 wheat farming households), and Asagirt (with 11,774 wheat farming households), were purposefully selected from the total of 11 administrative districts in the North Shewa Zone of the Amhara region. These districts were selected based on information accessed from the North Shewa Zone Agricultural Office, as they were identified to have active engagement in the wheat cluster farming initiative promoted by ATA.

In the second stage, three kebeles (villages) were randomly selected from each of the sampled districts, comprising two villages where the cluster farming initiative was actively implemented and one village where the initiative has not been adopted yet. This 2:1 ratio was adopted to allow for the comparative analysis between adopters and nonadopters of cluster farming while ensuring balanced representation across the districts.

In the third stage, farming households were randomly selected from each of the 12 sampled villages. A total sample of 398 households was determined using Yamane′s [45] formula on the equation below with a 95% confidence interval and a 5% margin of error, based on the total population of 95,950 wheat‐producing farming households across the four sampled districts.

Following probability proportional to size (PPS), in order to improve representativeness and minimize sampling bias, the sample was distributed proportionally to the number of wheat‐producing households in each sampled village. The final sample includes 249 adopters and 149 nonadopters of cluster farming. The table that follows presents the list of sampled villages, their participation status, total wheat farming households, and the number of sampled households per village.

n=N1+Ne2=95,950195,950+0.052=398.

In the equation above, *n* is the sample size, *N* is the total number of households, and *e* is the error tolerance or margin of error, which we have fixed at 5%. In the analysis of the study, four of the observations were not included due to missing data. Table 1 presents the distribution of farming households, village‐level cluster participation, and sample sizes across the selected districts.

**Table 1:** Sample size distribution by district and village.

**District/woreda**	**Farming household size per district**	**Village/kebele**	**Implementation of cluster**	**Farming household size per village**	**Sampled household size per village**
Siyadebirna Wayu	15,412	Siya Debre	Participant	1516	30
		Wele Denbiya	Participant	1529	30
		Gozegoza	Nonparticipant	866	25
Moretna Jiru	29,260	Mangudo	Participant	1517	30
		Woyira Amba	Participant	1079	21
		Bolo	Nonparticipant	1889	54
Mida Weremo	31,567	Remana Dire	Participant	2117	42
		Kera Mejit	Participant	2397	47
		Werke	Nonparticipant	1493	43
Asagirt	11,774	Seka	Participant	1118	22
		Gola	Participant	1343	27
		Wona	Nonparticipant	948	27

*Note:* The farming household size in sampled villages and districts was accessed from the North Shewa Zone Agricultural Office, and the sample size distribution was determined by the authors.

Structured questionnaire was used to collect data on the socioeconomic and demographic factors, institutional factors, plot characteristics, cluster farming and irrigation practices, wheat crop productivity, and food consumption expenditures of the sampled households. Prior to the full survey, a preliminary assessment was conducted to observe the state of smallholder farmers′ adoption of cluster farming to pretest the survey instruments and make adjustments accordingly. Before the data collection, the researchers made sure the enumerators had prior experience in data collection and they had provided them training on the survey questionnaire of the study. Furthermore, throughout both the pilot survey and the data collection process, the researchers collaborated with local agricultural and natural resource officers to supervise the data collection that spanned from June 2023 to December 2023.

### 3.3. Ethical Considerations

The researchers of this study have taken several ethical considerations throughout the study. A structured questionnaire survey was conducted among smallholder wheat farmers. Prior to commencing the study, the Research and Examination committee of Debre Berhan University has thoroughly reviewed and approved the study′s methodology and survey instruments. To ensure the consent of the respondents, the purpose of the research was clearly explained to all respondents before filling out the questionnaire or involving them either in interviews or focused group discussions. Consequently, informed consent was obtained in written form from all participants prior to their involvement in the study. The confidentiality of all personal data was strictly maintained throughout the research process.

### 3.4. Empirical Strategy

To meet the study′s objectives, a two‐phase analysis was conducted. The first phase was used to examine the determining factors of households′ decision to adopt cluster farming. The second phase used ESR to investigate the impact of cluster farming on farming households′ wheat productivity and welfare. Table 2 presents a description of the key explanatory variables used in this two‐phase analysis. Given its reliability as an indicator of households′ welfare, monthly per capita food expenditure was used as a proxy for households′ well‐being. To estimate this, respondents were asked to recall all food items consumed by the household over a 7‐day recall period, including items that were produced, purchased, or received as gifts or donations. Then, the quantities consumed were converted in to monetary values using average local market prices of the respective items within the study area.

**Table 2:** Description and measurement of variables and hypotheses for the selection variables.

	**Description and measurement**	**Expected sign**
Explanatory variables^a^		
Gender	Gender of the household head (female as reference)	±
Literacy	Literacy of the household head (illiterate as reference)	+
Age	Household head′s age	±
Marital status	Marital status of the head (unmarried as reference)	±
Experience	Household head′s experience in cluster farming (in years)	±
Household size	Total number of household members	+
Farm size	Size of land used for wheat cultivation (in hectare)	+
TLU	Tropical livestock unit (TLU)	+
Off‐farm activities	If the household is engaged in off farm activities	±
Access to credit	If the household has access to credit	+
Land certificate	If the household has land certificate	+
Extension contact	If the farm household has access to extension service	+
Farm cooperative	If the household is member of farms′ union	+
Access to information	If the household has information about cluster farming	+
Perceived impact	If the household perceives adopting cluster farming will impact their production positively	+
Training	If the household received training about cluster farming	+
Fertilizer	If the household used fertilizer	+
Irrigation	If the household used irrigation in cluster wheat production	+
Herbicide	If the household used herbicide	+
Improved seed	If the household used improved seed	+
Distance from market	Distance to the nearest market (in kilometer)	−
Distance from the plot	Distance from homestead (in kilometer)	−
Distance from road	Distance to the neatest all weather road (in kilometer)	−
Soil quality	Soil fertility: 1 if fertile, 2 if moderate, and 3 if degraded	−
Outcome variable		
Yield of wheat	Wheat yield in quintals per hectare (Q/hec) produced during 2023/2024 of Meher season	
Per capita consumption	Household′s per capita consumption expenditure per month (include all food and nonfood expenditure spend during the survey period)	

^a^Variables are selected based on the works of Abate [25], Hussen and Geleta [46], and Wogderes [47].

#### 3.4.1. ESR Model

Accurately measuring the impact of cluster farming on wheat productivity and household welfare requires addressing key econometric challenges such as unobserved heterogeneity, selection bias, and endogeneity. Many previous studies have relied on methods like OLS, PSM, IPWRA, and basic descriptive statistics to estimate the effects of agricultural technology adoption [25, 26, 29]. However, these approaches often fall short in constructing adequate counterfactuals and therefore may not capture the true causal impact of adoption [48, 49]. Moreover, estimating the effect of technology adoption is particularly challenging due to self‐selection, which is influenced by both observable and unobservable factors [50]. To overcome these limitations, this study employs a more robust approach, ESR that explicitly accounts for selection bias and endogeneity, and allows for a more credible estimation of adoption effects under actual and counterfactual scenarios [51].

The ESR model specification employed in this study is derived from the works of Lokshin and Sajaia [51] and Dubin and McFadden [52]. As stated in Dubin and McFadden, [52], the ESR model follows two stages of estimation. In its first stage, ESR uses a selection model to evaluate the study unit′s decision to choose a particular practice/technology. In its second stage, it uses an OLS model with selectivity correction that is computed from the first stage to evaluate the impact of the decision to adopt on the outcome variable.

In theory, farmers are expected to adopt a given technology/practice if they expect it to maximize their utility. And given scarcity of resource, they will adopt the one that can improve their farm profit rather than their operation cost [22, 23]. However, utility to be gained from adopting cluster farming cannot be directly observed. Instead, a random utility model can be used to state the conditional probability of adopting the practice given the farmers′ decision [53]. Following Lokshin and Sajaia [51], Asmare et al. [4], and Degefu et al. [24], this study employs a random utility framework to model farmers′ decisions to participate in cluster farming. The model assumes that a farm household will decide to adopt cluster farming if the expected utility to be generated from it is greater than other alternatives. This means, any given rational farm household *i* with objective of maximizing an outcome *C*_*i*_ (productivity or welfare) will first compare the benefits of alternative practices (*j* and *z*), considering factors such as extension services, agricultural inputs, technologies, cooperation, and information supplied and gained by a collective action before adopting the one with the highest net benefit. Since, expected utility is unobservable, participation occurs when the anticipated utility from cluster farming (*U*_*j*_) exceeds that from nonparticipation (*U* *z*), that is, *U*_*j*_ − *U* *z* > 0.

As it is indicated on the equation below, the net benefit the farm household is expecting to generate from adapting cluster farming is a latent variable, $C_{ij}^{*}$, and it is a function of observed covariates, *X*_*i*_, that represent household′s demographic, socioeconomic, institutional, and farm‐specific characters and *μ*_*i* *j*_, an error term that accounts for unobserved characteristics. Hence, the selection equation for the *i* ^th^ household will be specified as the equation as follows:

(1) Cij∗=βkXi+μij; with Ci=10 if Cij∗>0 otherwise,

where $C_{ij}^{*}$ is a latent variable that represent if the household has adopted cluster farming and *C*_*i*_ is its observable counterpart that will be equal to 1 if a farmer is to adopt the practice and 0 otherwise; *X*_*i*_ are a set of independent variables that influence adoption of cluster farming and *μ*_*i* *j*_ is random disturbances.

As it is mentioned earlier, the second stage of ESR is used to estimate the impact of adoption on the outcome variable. But such analysis requires controlling for the possible correlation between the household′s decision to adopt the practice and the error term. Hence, in this study, following Lokshin and Sajaia [51], the productivity/welfare function of the two possible regimes (Regime 1 to adopt and Regime 0 not to adopt) is estimated separately while controlling for the endogenous nature of the adoption decision and selection bias (Equations (2a) and (2b)).

(2a) Regime 00:wP0=α0X0i+e0i if Cij=,

(2b) Regime 11:wP1=α1X1i+e1i if Cij=.

In the system of the equation above, *w* *P*_0_ and *w* *P*_1_ represent the possible production/welfare outcome during Regimes 0 and 1. *X*_*i*_ denote exogenous factors that are expected to influence wheat productivity and welfare, *α*_*i*_ are parameters to be estimated, and *e*_*i*_ denotes error terms. Finally, as indicated in Lokshin and Sajaia [51], the error terms of the selection equation and the productivity/welfare functions of the two regimes will have trivariate normal distribution, with zero mean and nonsingular covariance matrix expressed as

cove1,e0,μ=δe02.δe0μ.δe12δe1μ..δμ2,

where $\delta_{\mu }^{2}$ is the variance of the error term in the selection Equation (1) (which can be assumed to be equal to 1 since the coefficients are estimable only up to a scale factor), $\delta_{e_{0}}^{2}$ and $\delta_{e_{1}}^{2}$ are the variances of the error terms in the outcome functions (Equations (2a) and (2b)), and *δ*_*e*0*μ*_ and *δ*_*e*1*μ*_ represent the covariance of the error term of the selection equation (*μ*), and the outcome equation of Regime 0 (*e*_0_) and Regime 1 (*e*_1_). The covariance between *e*_0_ and *e*_1_ is not defined, as *C*_1_ and *C*_0_ are never observed simultaneously [54].

An important implication of the error structure is that, because the error term of the selection equation, *μ*, is correlated with the error terms of the productivity/welfare outcome functions (Equation (2a) and (2b)) (*e*_0_ and *e*_1_), the expected values of *e*_0_ and *e*_1_ conditional on the sample selection are nonzero. Mathematically,

Ee1Ci=1=δe1μϕβXiϕβXi=δe1μλ1=Ee0/Ci=0=−δe0μϕβXi1−ϕβXi=δe0μλ0,

where *ϕ*(.) is the standard normal probability density function, *ϕ*(.) is the standard normal cumulative density function, and *λ*_1_ = *ϕ*(*β*Xi)/*ϕ*(*β*Xi) and *λ*_0_ = *ϕ*(*β*Xi)/1 − *ϕ*(*β*Xi).

The inverse mills ratios computed from the selection equation are included in the outcome equation. If the estimated covariance *δ*_*e*1*μ*_ and *δ*_*e*0*μ*_ are statistically significant, then it indicates that the decision to adopt and the productivity/welfare outcome variables are correlated. This provides evidence of endogenous switching, rejecting the null hypothesis of no sample selectivity bias. Traditionally, the estimation of a model involving self‐selection has been done using a two‐stage procedure.

However, this method is considered inappropriate and it is highly criticized. It requires adjustments to derive consistent standard errors, and it performs poorly when there is high multicollinearity between the covariates of error terms of Equations (1), (2a), and (2b) [55]. As stated in Lokshin and Sajaia [51], in this case, a full information maximum likelihood (FIML) can be used for the endogenous switching model to generate appropriate and efficient estimates. Since the FIML method can simultaneously estimate the selection equation and regression equations, it can generate consistent standard errors. It is preferred over the two‐stage procedure as it can provide more robust and efficient estimates while accounting for the potential endogeneity and sample selectivity issues. Assuming a trivariate normal distribution for the error terms, the logarithmic likelihood function for the system of Equations (1), (2a), and (2b) can be specified as:

LnLi=∑i−1NCilnϕe1δe1−lnδe1+lnϕτ1i+1−Cilnϕe0δe0−lnδe01−lnϕτ0i,

where $\tau_{ji}=\langle \beta Xi+p_{j}e_{ji}/\delta_{j}/\sqrt{1-p_{j}^{2}}\rangle ,j\;i=12,$, with *δ*_*j*_ denoting the correlation coefficient between the error term *μ* of the selection Equation (1) and the error term *e*_*j* *i*_ of Equations (2a) and (2b). After estimating the model′s parameters, the model calculate the expected outcomes (productivity/welfare) for both adopters and nonadopters of cluster farming and their potential counterfactual scenarios. In doing so, it generates expected productivity and welfare of adopters (a) and nonadopters (b), and for their hypothetical counter factual cases expected productivity and welfare levels had adopters did not adopt (c) and had nonadopters adopted the practice (d). And these values are used to estimate the average treatment effect (ATE) of adopting cluster farming as shown on the equations below.

The conditional expected values of the outcome variables of this study during the four scenarios mentioned above will be:

Case 1: The farm household adopts cluster farming (actual)

(3a) EwP1C=1=α1X1+δe1μλ1.

Case 2: The farm household did not adopt cluster farming (actual)

(3b) EwP0C=0=α2X0+δe0μλ0.

Case 3: Adopters had they decided not to adopt (counter factual)

(3c) EwP1C=0=α1X0+δe1μλ0.

Case 4: Nonadopters had they decided to adopt (counter factual)

(3d) EwP0C=1=α2X1+δe0μλ1.

Using the four equations above, the ATE of adopting cluster farming on the outcome variables will be generated by finding the difference between the actual expected outcome when the farm household decides to adopt (3a) and the counter factual expected outcome had nonadopters decided to adopt (3d).

(4) ATT=EPw1C=1−EwP0C=1=X1α1−α2+λ1δe1μ−δe0μ.

And the average treatment effect on the untreated (ATU) for the farm households that did not adopt cluster farming will be the difference between the expected outcome when the farm household did not adopt and the counter factual expected outcome had adopters decided not to adopt cluster farming (3b and 3c).

(5) ATU=EwP1C=0−EwP0C=0=X0α1−α2+λ0δe1μ−δe0μ.

Finally, the transitional heterogeneity (TH) which indicates whether the effect of adopting cluster farming is higher for the adopters compared to the nonadopters is computed by finding the difference between the treatment effect on treated (TT) and the treatment effect on the untreated (TU).

#### 3.4.2. Model Identification Test

Following Di Falco and Veronesi [56], this study applies an exclusion restriction test to check if proper identification of ESR was used. The test works by excluding the explanatory variables selected to affect the selection equation (decision to adopt cluster farming) directly but not the outcome equation (productivity/welfare equation) from the outcome equation. Since the Mills ratio is a nonlinear function of the explanatory variables in the selection equation and testing for nonlinearity is complex, other variables that affect the selection but not the outcome were supposed to be selected. For these variables to be considered valid instruments, it must be proven (either theoretically or empirically) that they influence the adoption decision of the farm household but not its wheat production or welfare. Therefore, to confirm the validity of the model, we used variables such as access to cluster information, perceived benefits of cluster farming, training access, and distance to farm (for the consumption equation) as selection instruments. The validity of the instrumental variables was empirically tested using a falsification test, which checks for the presence of correlation between the instruments and the unobservable factors. As indicated in the appendix, the Wald test for the exogeneity of the selection instruments shows high insignificance at 5% indicating their validity.

## 4. Results and Discussion

### 4.1. Descriptive Statistics

Table 3 presents the descriptive statistics of explanatory and outcome variables used in the study and shows if there is a statistically significant difference between adopters and nonadopters of cluster wheat production in the study area. The study initially sampled 398 farm households. After excluding four households with missing data in key variables, the final sample used for empirical analysis consisted of 394 households. Among these, 62% of them are found to produce wheat in cluster farming. The average age of the households is 43, and most of them are married males. The percentage of literate household heads is significantly higher among adopters of cluster farming. This is in line with the findings of Degefu et al. [24], who reported that cluster farming participants tend to be more educated than nonparticipants. The average household size of households that adopt cluster farming is 5, and it is significantly higher than the household size of the nonadopters. This suggests that a household with a larger household size may have greater labor availability, enabling them to manage the labor‐intensive requirements of cluster farming. The majority of the sampled households are found to have land certificates and have 20‐plus years of farming experience. On average, adopters of the practice are found to have significantly higher livestock but lower land size allotted for cluster farming. This aligns with the findings of Degefu et al. [24], who reported that cluster farming participants possess more livestock assets than nonparticipants, with the difference being statistically significant.

**Table 3:** Descriptive statistics of variables used in the regression.

**Selected variables**	**Nonadopters**	**Adopters**	**Both**	**p** **value**
Number of observation	149	245	394	
Distance to the main road (km)	1.49 (2.33)	1.83 (2.26)	1.70 (2.28)	
Distance to the nearest market (km)	6.73 (2.70)	4.96 (3.86)	5.63 (3.56)	^∗∗∗^
Gender of HH (if male)	125 (83.9%)	223 (91.0%)	348 (88.32%)	^∗∗^
Age of household head (years)	43.97 (10.75)	43.30 (9.86)	43.55 (10.19)	
Literacy of HH (if literate)	107 (71.8%)	198 (80.8%)	305 (77.41%)	^∗∗^
Marital status (if married)	115 (77.2%)	205 (83.7%)	320 (81.22%)	
Household size (in number)	4.48 (1.96)	5.12 (1.91)	4.87 (1.95)	^∗∗∗^
Farming experience (farming years)	26.15 (12.82)	24.48 (11.81)	25.11 (12.21)	
Land right certification (if certified)	131 (87.9%)	222 (90.6%)	353 (89.59%)	
Tropical livestock unit (TLU)	6.61 (3.34)	7.90 (5.20)	7.41 (4.62)	^∗∗∗^
Information access to cluster farming (if yes)	124 (83.22%)	243 (99.18%)	367 (93.15%)	^∗∗∗^
Perceived impact of cluster farming (if yes)	66 (44.30%)	226 (92.24%)	292 (74.11%)	^∗∗∗^
Off farm work engagement (if yes)	128 (85.91%)	220 (89.80%)	348 (88.32%)	^∗∗^
Training related to cluster farming (if yes)	95 (63.8%)	234 (95.5%)	329 (83.50%)	^∗∗∗^
Size of land allotted to produce wheat (hectare)	3.49 (2.12)	2.42 (1.52)	2.82 (1.84)	^∗∗∗^
Improved seed (if HH adopted)	106 (71.1%)	208 (84.9%)	314 (79.70%)	^∗∗∗^
Fertilizer (if HH adopted)	128 (85.9%)	220 (89.8%)	348 (88.32%)	
Herbicide (if HH adopted)	70 (47.0%)	180 (73.5%)	250 (63.45%)	^∗∗∗^
Fertile soil quality	23 (15.4)	73 (29.8)	96 (24.37)	^∗∗∗^
Moderate soil quality	118 (79.2)	162 (66.1)	280 (71.07)	^∗∗∗^
Degraded soil quality	8 (5.4)	10 (4.1)	18 (4.56)	
Irrigation (if HH adopted irrigation)	38 (25.5%)	102 (41.6%)	140 (35.53%)	^∗∗∗^
Farmers′ union (if HH is member)	110 (73.8%)	210 (85.7%)	320 (81.2%)	^∗∗^
Credit (if HH has access)	88 (59.1%)	151 (61.6%)	239 (60.66%)	
Extension contact (if HH has access)	133 (89.3%)	244 (99.6%)	377 (95.69%)	^∗∗∗^

*Note:* The figures in parentheses represent standard deviations for continuous variables and percentages for categorical variables. The mean comparison test (*t*‐test) and chi‐squared test (indicated by the *p* values) were used to compare the means and percentages of explanatory variables between nonadopters and adopters of cluster farming.

^∗∗^ and  ^∗∗∗^ denote significance level at 5% and 1%, respectively.

Compared to nonadopters, a significantly higher proportion of adopters is found to have access to information on cluster farming, access to extension service, engagement in off‐farm activities, received training on cluster farming, and use irrigation in production. These results are in line with previous research. Amir et al. [57] noted that access to information empowers farmers to make informed decisions regarding crop and variety selection. Abate [25] emphasized the role of training and extension services in enhancing farmers′ understanding of cluster farming practices and accelerating adoption. Similarly, the ATA [44] highlighted that access to irrigation not only supports the implementation of cluster farming but also leads to increased productivity, more efficient resource use, and higher profitability. Furthermore, Zegeye et al. [22, 23] found that households engaged in off‐farm activities are more likely to adopt cluster farming, as they benefit from increased financial resources, reduced risks, exposure to new ideas, and enhanced decision‐making capacity.

Again, compared to nonadopters, a significantly higher proportion of adopters is found to use modern farm inputs like improved seed and herbicide in their production of wheat. This aligns with De Janvry et al. [58], who found that access to modern technologies enhances productivity, improves efficiency, optimizes resource use, and strengthens market integration in cluster farming. In this study, soil fertility on households′ land holdings is measured using a categorical variable where 1 is given for fertile, 2 is given for moderate, and 3 is given for degraded quality soil. As a result, outputs of the descriptive statistics indicate that, compared to nonadopters, a higher proportion of adopters of cluster farming have good soil quality, while fewer adopters have fair or poor soil quality. The differences are statistically significant for good and fair soil categories, suggesting that adopters generally cultivate plots with relatively better soil fertility. Finally, the average distance from home to all‐weather roads is higher for adopters, but from home to markets is greater for nonadopters than for their counterparts. This is consistent with Ahmed et al. [59] and Zegeye et al. [41], who found that farmers farther from all‐weather roads and market centers are less likely to adopt new technologies due to limited information access, delayed adoption, and higher production costs, a difference that was statistically significant between the groups.

To sum up, the mean comparison above indicates the presence of heterogeneity between adopters and nonadopters of cluster farming. This indicates that to help the nonadopters adopt cluster farming in wheat production, investments are needed so that it is possible to create awareness by providing training services about the applicability and benefits of cluster farming; expand access to infrastructure like roads, markets, and service centers; and provide access to irrigation and modern inputs.

### 4.2. Econometric Result

Tables 4 and 5 present the outputs of the FIML and ESR model estimations. Before discussing the results, the joint independence of the three equations—one selection and the two output equations—was checked, and the result indicates the rejection of the null hypothesis at the 1% level of significance indicating unobserved factors jointly influence both selection into cluster farming and resulting outcome variables. The presence of selection bias was tested using the correlation coefficient (rho) between the error term of the selection and outcome equations. Rho is found to be negative and statistically significant, indicating the rejection of the null hypothesis of no selection bias. This confirms the presence of selection bias in the adoption of cluster farming and justifies the use of the ESR model. Furthermore, as can be seen from Tables 4 and 5, the sign of rho is negative both for the adopters and the nonadopters. This indicates that, compared to a randomly selected farm household, those who adopt cluster farming have significantly higher wheat production and per capita consumption expenditure. The reverse holds true for the nonadopters. Finally, the falsification test presented in Tables A1 and A2 in the appendix confirms the validity of the selected instrumental variables and indicates the model is adequately identified.

**Table 4:** FIML estimate of the endogenous switching regression model for wheat productivity.

**Variables**	**Adoption (1/0)**		**Wheat productivity for adopters**		**Wheat productivity for nonadopters**	
	**Coeff**	**SE**	**Coeff**	**SE**	**Coeff**	**SE**
Gender	0.401	0.258	−0.709	0.240	0.272 ^∗∗^	0.138
Age	−0.045 ^∗∗∗^	0.013	0.354	0.317	0.176	0.345
Literacy	0.493 ^∗∗^	0.206	2.122 ^∗∗∗^	0.414	0.968 ^∗∗^	0.384
Marital	−0.167	0.225	0.549 ^∗∗∗^	0.100	−0.800 ^∗∗^	0.402
Experience	−0.034 ^∗∗∗^	0.010	0.289	0.237	−0.414 ^∗∗^	0.185
Land right	0.085	0.301	−0.461	0.301	−0.331	0.219
Wheat farm size	0.000	0.076	0.723 ^∗∗∗^	0.135	0.443 ^∗∗∗^	0.149
Herbicide	0.061	0.291	0.818	0.550	0.964	0.594
Moderate soil quality	−0.475 ^∗∗^	0.225	−0.068	0.934	−0.845	0.919
Degraded soil quality	−0.293	0.427	−0.246	0.164	−0.690	0.610
Irrigation access	0.197 ^∗∗^	0.101	1.957 ^∗∗^	0.786	−0.464	0.695
Credit access	0.057	0.175	−0.527	0.915	0.306 ^∗∗∗^	0.105
Cooperative	0.388 ^∗∗∗^	0.033	0.841	0.941	1.255 ^∗∗∗^	0.300
TLU	0.005	0.023	0.155	0.471	1.434 ^∗∗^	0.643
Distance to road	−0.057	0.046	−1.018	0.768	−1.281 ^∗^	0.776
Distance to market	−0.075 ^∗∗^	0.034	−1.358 ^∗^	0.777	0.673	0.952
Distance to plot	−0.228 ^∗^	0.123	−0.760	0.334	0.821	0.526
Household size	0.053	0.062	0.129 ^∗∗^	0.053	0.708	1.212
Fertilizer	0.639 ^∗∗∗^	0.206	0.499 ^∗∗∗^	0.141	0.695 ^∗^	0.414
Off farm work	0.175	0.255	−0.850 ^∗^	0.486	−0.765 ^∗^	0.416
Improved seed	0.203 ^∗∗∗^	0.064	0.244 ^∗∗∗^	0.040	0.388	0.554
Extension	0.531	0.501	0.427	0.343	0.785	0.514
Cluster information	0.302	0.387				
Perception	1.317 ^∗∗∗^	0.260				
Training	0.315 ^∗∗∗^	0.103				
_cons	−3.179	1.206	−7.448	3.693	9.004	15.211
/lns1	2.935	0.014				
/lns2	2.672	0.081				
/r1	0.075	0.515				
/r2	−0.474 ^∗∗^	0.228				
*N* *u* *m* *b* *e* *r* *of* *o* *b* *s* = 394 *W* *a* *l* *d* ^′^ *s* *t* *e* *s* *t* *of* *i* *n* *d* *e* *p*.*e* *q* *n* *s*.:*p* *r* *o* *b* > *c* *h* *i* ^2^ = 0.0000 *W* *a* *l* *d* ^′^ *s* *t* *e* *s* *t* *of* *m* *o* *d* *e* *l* *a* *d* *e* *q* *u* *a* *c* *y* : *p* *r* *o* *b* > *c* *h* *i* ^2^ = 0.0000						

*Note: Source:* Authors′ estimates (2024).

^∗^,  ^∗∗^, and  ^∗∗∗^ denote 10%, 5%, and 1% levels of significance, respectively.

**Table 5:** FIML estimate of the endogenous switching regression model for per capita consumption expenditure.

**Variables**	**Adoption (1/0)**		**Per capita consumption expenditure for adopters**		**Per capita consumption expenditure for nonadopters**	
	**Coeff**	**SE**	**Coeff**	**SE**	**Coeff**	**SE**
Gender	0.369	0.267	−0.555	0.534	0.264 ^∗∗∗^	0.792
Age	−0.044 ^∗∗∗^	0.013	−0.010	0.014	0.018	0.032
Literacy	0.471 ^∗∗^	0.212	0.063	0.229	0.348	0.255
Marital	−0.153	0.247	0.780 ^∗∗^	0.351	2.165 ^∗∗∗^	0.689
Experience	−0.033 ^∗∗∗^	0.010	0.015	0.011	2.264	0.792
Land right	0.038	0.301	0.854	0.660	0.018	0.032
Wheat farm size	0.227 ^∗∗∗^	0.067	0.076 ^∗∗∗^	0.021	0.348	0.255
Herbicide	0.045	0.295	−0.007	0.328	−0.165	0.689
Moderate soil quality	−0.493 ^∗∗^	0.206	−0.229	0.222	−0.319	0.212
Degraded soil quality	−0.274	0.419	−0.092	0.473	−0.761	0.531
Irrigation access	0.127	0.197	0.382 ^∗^	0.213	0.335 ^∗∗^	0.124
Credit access	0.072	0.169	0.409 ^∗∗^	0.186	0.462	0.358
Cooperative	0.159	0.365	1.445 ^∗∗^	0.667	0.431	0.517
TLU	0.005	0.023	0.174 ^∗∗∗^	0.028	0.186 ^∗∗∗^	0.060
Distance to road	−0.056	0.045	−0.137 ^∗∗^	0.064	−0.148 ^∗∗^	0.068
Distance to market	−0.082 ^∗∗^	0.032	−0.033	0.045	−0.140	0.100
Household size	0.059	0.062	−0.700 ^∗∗∗^	0.077	−0.778 ^∗∗∗^	0.161
Fertilizer	0.694 ^∗∗∗^	0.209	0.367	0.282	0.271	0.322
Off farm work	−0.193	0.259	0.329	0.310	−0.619	0.396
Improved seed	−0.060	0.210	0.038	0.243	0.124	0.262
Extension	0.721	0.514	0.116	0.494	0.193	0.286
Distance to plot	−0.256 ^∗∗^	0.114				
Cluster information	0.393 ^∗∗∗^	0.122				
Perception	1.172 ^∗∗∗^	0.242				
Training	0.255	0.339				
_cons	−2.895 ^∗∗^	1.178	8.789 ^∗∗∗^	1.754	5.284 ^∗∗∗^	1.167
/lns1	0.321 ^∗∗∗^	0.089				
/lns2	0.454 ^∗∗∗^	0.090				
/r1	−0.051	0.174				
/r2	−0.092 ^∗∗∗^	0.013				
*N* *u* *m* *b* *e* *r* *of* *o* *b* *s* = 394 *W* *a* *l* *d* ^′^ *s* *t* *e* *s* *t* *of* *i* *n* *d* *e* *p*.*e* *q* *n* *s*.:*p* *r* *o* *b* > *c* *h* *i* ^2^ = 0.0354 *W* *a* *l* *d* ^′^ *s* *t* *e* *s* *t* *of* *m* *o* *d* *e* *l* *a* *d* *e* *q* *u* *a* *c* *y* : *p* *r* *o* *b* > *c* *h* *i* ^2^ = 0.0000						

*Note: Source:* Authors′ estimates (2024).

^∗^,  ^∗∗^, and  ^∗∗∗^ denote 10%, 5%, and 1% levels of significance, respectively.

#### 4.2.1. Determinants of Household′s Decision to Adopt Cluster Farming

Before interpreting the outputs of the selection equation, various postestimation tests were undertaken to check for the validity of the results. The model adequacy test rejects the null hypothesis at 1% (*p* > 0.000), indicating the coefficients are jointly significant. Robust standard errors were used to control for possible heteroskedasticity and nonnormality problems.

The result shows that the household head′s age negatively affects the probability of adopting cluster farming, indicating that younger household heads are more likely to adopt the practice than older ones. This could be because younger heads are more likely to receive formal education and, as indicated in Teklewold [60], tend to be less risk‐averse. The finding aligns with Teklewold [60], Hussen and Geleta [46], and Ayenew et al. [61].

The literacy level of the household head positively influences the likelihood of adopting cluster farming, showing that educated households are more likely to adopt the practice than nonadopters. This is because educated farmers are better able to acquire, analyze, and evaluate information about cluster farming methods, market opportunities, and associated benefits. Studies like Abate [25] indicate a positive correlation between education and farmers′ probability of adopting modern farm technologies and practices. Similarly, Adebayo [62], Feyisa [63], and Abate [25] found that educated household heads are better equipped to acquire, analyze, and assess information on modern technology and market opportunities, supporting this finding.

Farming experience has a negative and significant effect on the probability of adopting cluster farming, suggesting that households with more farming experience are less likely to adopt cluster farming than their less‐experienced counterparts. This is consistent with Alhassan et al. [64] and Hussen and Geleta [46], which indicate that extensive farming experience increases farmers′ risk‐averting behavior, reducing their likelihood of adopting modern technologies and practices.

The household head′s membership in a farmers′ union positively influences the probability of adopting cluster farming more than the nonmembers. This is because farmers′ unions have the ability to make information on price and new technologies readily available and modern farm inputs easily accessible. As a result, farmers whose heads are members of such unions are expected to be more likely to adopt modern farm technologies and practices. Moreover, as indicated in Amir et al. [57], access to such information increases farmers′ awareness and knowledge about cluster farming, including its benefits and costs, and even helps them make informed decisions regarding what crops and crop varieties to plant. This finding aligns with Nyangau et al. [65], Abate [25], and Wogderes [29].

The household head′s perception of cluster farming significantly influences the probability of adopting the practice. Households that perceive positive effects from cluster farming are more likely to adopt it than those who do not. Factors such as knowledge, perceived benefits, risk perception, social influence, and institutional support play an important role in shaping household perceptions about a particular technology or practice. Consistent with Hussen and Geleta [46] and Abate [25], positive perception by farmers can increase the likelihood of adopting a technology or practice by addressing their concerns and building trust in its benefits, thereby encouraging informed decision‐making and willingness to adopt modern farming practices.

Access to fertilizer and improved seed significantly increases the probability of adopting cluster farming in the study area. Access to modern agricultural technologies, such as fertilizer and improved seed, enhances cluster farming by improving efficiency and productivity, thereby ensuring the profitability and sustainability of the practice. These findings are supported by ATA [44], Wogderes [29], and De Janvry et al. [58], which indicate that access to modern inputs enables farmers to achieve higher yields, better resource‐use efficiency, and more sustainable farming outcomes.

Farmers who have received training on cluster farming have a positive effect on the likelihood of adopting the practice. The positive effect of training can be attributed to its ability to improve farmers′ capacity to receive, decode, and understand relevant information, enabling them to make informed and appropriate decisions. The finding is similar to Ayenew et al. [61] and Abate [25], which indicate that providing training on cluster farming can play a critical role in expanding the practice among smallholder farmers, enhancing their knowledge, skills, and adoption of modern farming techniques.

The wheat farm size of a farm household has a positive and significant effect on the probability of adopting cluster farming in the study area. Parallel to the works of Alwang et al. [66], Wogderes [29], and Feyisa [63], households with larger land holdings are more likely to adopt cluster farming as their primary source of livelihood. Studies by Feyisa [63] indicate that as households′ land holdings increase, they are more motivated to make long‐term investments in their land and adopt modern agricultural technologies. The finding is consistent with Alwang et al. [66], Wogderes [29], and Feyisa [63], suggesting that larger land size allotted to wheat production positively influences the probability of adopting cluster farming in the study area.

The other findings of this study showed that, households with plots of moderate soil quality are less likely to adopt cluster farming compared to those with fertile soil. This indicates that relatively poorer soil conditions may discourage adoption, as farmers perceive that the benefits from cluster farming on such land would be limited. This result falls in a similar line to Abate [25] and Ayenew et al. [61], which show that farmers are more likely to adopt a practice or technology when they expect higher returns from it.

Access to irrigation has a positive and significant effect on the probability of adopting cluster farming. Irrigation provides a reliable water supply for crops, including during dry seasons, which facilitates the adoption of winter cluster farming. By ensuring water availability and mitigating risks associated with erratic rainfall, irrigation plays a crucial role in enhancing the adoption of cluster farming and other modern agricultural technologies and practices. The finding is parallel with Dureti et al. [35] and ATA [44], which indicate that households with access to irrigation are more likely to invest in cluster farming, resulting in improved yields, greater resource‐use efficiency, and higher profitability.

Households′ distance from their farm plots negatively influences the probability of adopting cluster farming. This is because the farther a household is from its plot, the less likely it is to invest in plot preparation and other modern farm inputs, which reduces the likelihood of adopting cluster farming. The finding is in line with Zegeye et al. [22, 23] and Tefera et al. [67], indicating that proximity to farm plots plays an important role in facilitating investment in modern farming practices and technology adoption.

Finally, farm households′ proximity to markets is found to significantly improve their probability of adopting cluster farming. This is due to the fact that households located closer to market centers are more likely to adopt new farming techniques and technologies because they have better access to information on improved technologies, modern inputs, and markets for their outputs. Conversely, farmers living farther from market centers may face delays in adoption, higher production costs, and limited access to relevant information, reducing their likelihood of adopting modern farming practices. The finding is similar to Ahmed et al. [68], which shows that in developing economies, smallholder farmers who are located near markets tend to adopt new technologies and production techniques more quickly than those living farther away.

#### 4.2.2. Determinants of Wheat Productivity and Per Capita Consumption

As shown in Table 4, the outputs of the OLS regression indicate smallholder farmers′ wheat productivity is influenced by different factors for adopters and nonadopters of cluster farming. Gender has a negative but insignificant effect on adopters′ productivity, while it is positive and significant for nonadopters. Marital status, livestock holdings (TLU), and access to irrigation positively and significantly enhance productivity for both groups, whereas household size and distance to the nearest road negatively and significantly affect productivity. For adopters, additional significant positive factors include wheat farm size, access to credit, and cooperative membership. Age shows different signs for adopters and nonadopters but is not significant for either group.

With regard to the determinants of households′ per capita food consumption expenditure per month, as shown in Table 5, the effects differ between adopters and nonadopters of cluster farming. Gender has a negative but insignificant effect on adopters′ per capita consumption, whereas it is positive and significant for nonadopters. Marital status, livestock holdings (TLU), and access to irrigation positively and significantly enhance consumption expenditure for both groups, while household size and distance to the nearest road have negative and significant effects. For adopters, additional significant positive factors include wheat farm size, access to credit, and cooperative membership. Age shows different signs for adopters and nonadopters but is not significant for either group. These variations, particularly in the sign and significance of gender and age, indicate heterogeneity between adopters and nonadopters in determining per capita consumption expenditure. And, like the case of wheat productivity, some explanatory variables are found to have opposite signs and significance for adopters and nonadopters. These variations highlight the heterogeneity between adopters and nonadopters, consistent with the findings of Di Falco et al. [69] and Khanal et al. [70].

#### 4.2.3. Impact of Cluster Farming on Wheat Productivity

As shown in Table 6, the impact of adopting cluster farming on wheat yields is represented by the ATE and it is determined by finding the difference between actual and counter factual average wheat yield per hectare. The results indicate both the average treatment effect on treated (ATT) and ATU are positive and statistically significant. Specifically, the average wheat productivity of the adopters was 4234.8kg/ha. But had these same households been nonadopters of cluster farming, their yield could have been only 3395.5 kg/ha which is an 839.3 kg/ha reduction. This difference represents the ATT and demonstrates that, by adopting cluster farming, households causally increase their wheat yields by 839.3 kg/ha, controlling for selection bias and household characteristics. On the other hand, the average wheat productivity of nonadopters of cluster farming was 3801.7 kg/ha. But if these same farmers were adopting clustered wheat production, their average yield could have been 4439 kg/ha which is higher by 637.3 kg/ha. This reinforces the causal effect of cluster farming, showing that even households that have not adopted the practice could significantly improve wheat yields if they implement it. Based on these results, we can say that in this study area, adopting cluster farming can make a significant and positive impact on farmers′ wheat yield.

**Table 6:** Estimation of conditional expectations, treatment, and heterogeneity for wheat productivity measured in kilograms per hectare.

**Alternative**	**Decision stage**		**ATE**
	**To adopt**	**Not to adopt**	
Adopters	4234.8 (97.3)	3395.5 ^″^ (27.2)	TT = 839.3^∗∗∗^
Nonadopters	4439.0 ^″^ (27.6)	3801.7 (59.3)	TU = 637.3^∗∗^
TH effect			TH = 201.9^∗∗^

*Note:* Standard errors are in parentheses, and  ^∗∗^ and  ^∗∗∗^ denote significance levels at 1% and 5%, respectively. *Source:* Authors′ estimates (2024).

Abbreviations: TH (TT‐TU), transitional heterogeneity; TT, adoption effect for adopters; TU, adoption effect for nonadopters.

The TH, computed as the difference between ATT and ATU, is positive and statistically significant, indicating that households who are current adopters have a higher advantage in improving their wheat yield by continuing to implement the practice relative to nonadopters, highlighting the differential gains from cluster farming based on adoption status.

#### 4.2.4. Welfare Impacts of Adopting Cluster Farming

Table 7 presents the actual and counterfactual average monthly per capita food consumption expenditure and the ATT and ATU generated from it. As shown on the table, the ATT which is the difference between the average monthly per capita food expenditure of adopters and the average that could have been had these same households been nonadopters is found to be $12.49 (=birr 698.85)^1^. This demonstrates that by adopting cluster farming for their wheat production, households have increased their per capita food consumption by $12.49, highlighting the positive impact of this agricultural practice. This finding indicates that adopting cluster farming causally improves household welfare, as it directly contributes to higher per capita consumption.

**Table 7:** Estimation of conditional expectations, treatment, and heterogeneity effect for per capita monthly consumption expenditure in dollars.

**Alternative**	**Decision stage**		**Adoption effect**
	**To adopt**	**Not to adopt**	
Adopters	59.97 (0.0017)	47.48 ^″^ (0.0021)	TT = 12.49^∗∗∗^
Nonadopters	62.14 ^″^ (0.0026)	42.91 (0.0023)	TU = 19.23^∗∗∗^
TH effect			TH = −6.75^∗∗^

*Note:* Standard errors are found in the parentheses, and  ^∗∗∗^,  ^∗∗^,  ^∗^ denote the level of significance at 1%, 5%, and 10%, respectively. *Source:* Authors′ estimates (2024).

Abbreviations: TH (TT‐TU), transitional heterogeneity; TT, adoption effect for adopters; TU, adoption effect for nonadopters.

On the other hand, the ATU which is the difference between average per capita monthly food expenditure of nonadopters and the average that could have been had these same households adopted the practice is found to be $19.23. These show that the households could have improved their welfare status by adopting the practice. Furthermore, as indicated by the negative and significant TH, compared to the adopters, in this study area, the impact of cluster farming is higher for households that are currently not adopting the practice. This indicates there is a significant advantage for nonadopting households to improve their welfare by adopting the cluster farming practice. By using the ESR model, this study corrects for self‐selection and unobserved heterogeneity, allowing us to attribute observed differences in productivity and welfare directly to the adoption of cluster farming rather than confounding factors. The model′s identification strategy, which relies on instrumental variables to account for factors influencing adoption but not directly affecting productivity, strengthens the causal interpretation of the results.

Overall, the results of the impact analysis confirm that adopting cluster farming significantly increases wheat yields and household per capita food consumption. That is, the farmers who actually adopt cluster farming would have experienced lower wheat yields and welfare if they had not adopted, while the farmers who did not adopt would have gained higher benefits if they had adopted the practice. Additionally, the analysis reveals the presence of heterogeneity in the impacts. As it can be seen from the tables, the counterfactual wheat yields of the nonadopter households are higher than the actual yields of households that are currently adopting the practice. This suggests the nonadopters of the practice have systematically different household, plot, and institutional characteristics compared to the adopters. These findings are consistent with previous studies ([4, 25, 26, 30]; [32] [29, 31]) and reinforce the importance of promoting cluster farming as a policy for improving both agricultural productivity and household welfare.

## 5. Conclusion and Recommendations

This study examined the impact of cluster farming adoption on wheat productivity and household welfare in the North Shewa Zone of the Amhara region, Ethiopia. Using data from 394 farm households across four districts and applying an ESR model, the study assessed both the determinants of adoption and its effects. The findings revealed that the likelihood of adopting cluster farming was positively and significantly influenced by factors such as the education level of the household head, farm size, access to irrigation, the use of modern farm inputs, farmers′ perception of the benefits of cluster farming, membership in farmers′ unions, and access to training and information. Conversely, adoption was negatively and significantly affected by the age of the household head, farming experience, distance to plots, poor soil fertility, and distance to markets.

The impact analysis showed that cluster farming adoption led to significantly higher wheat yields among adopters. Furthermore, the counterfactual analysis indicated that nonadopters could have achieved higher wheat productivity had they adopted the practice. In addition to productivity gains, the study found that cluster farming significantly increased per capita food consumption expenditure for both adopters and potential adopters. This suggests that cluster farming not only enhances productivity but also contributes to improving household welfare through better food consumption.

Based on these findings, the study recommends that the government and other stakeholders collaborate with rural farming households to promote the expansion of cluster farming and maximize its benefits for smallholder farmers. Although adoption has shown positive results, the relatively low participation rate suggests the need for targeted, context‐specific interventions. Since access to training, extension services, literacy, and information was found to positively influence adoption, strengthening farmer training programs and integrating cluster farming into rural extension and adult education services is essential. Efforts should also focus on improving infrastructure, such as rural roads and irrigation systems, and ensuring the timely distribution of quality inputs to address the barriers posed by market distance and input shortages.

Furthermore, the positive role of farmers′ unions in adoption highlights the importance of collective access to inputs, knowledge sharing, and market linkages. Therefore, the development and strengthening of inclusive, well‐functioning unions, especially in low‐adoption areas, should be prioritized. Additionally, because poor soil fertility was associated with lower adoption rates, targeted soil improvement programs should be implemented to enhance the feasibility of cluster farming for affected households. Lastly, the study recommends stronger collaboration between farmers and key stakeholders, including research institutions, agricultural offices, and development partners, to ensure the effective transfer of innovations, better resource utilization, and the long‐term sustainability of the cluster farming initiative.

## 6. Limitations of the Study

This study has a few important limitations. First, the analysis is based on cross‐sectional data, which restricts the ability to observe changes over time or establish strong causal relationships. Using panel data in future research would allow for a more comprehensive understanding of the dynamics of cluster farming adoption and its long‐term impacts on productivity and welfare. Second, the study was conducted in a single zone, North Shewa of the Amhara region, which may limit the generalizability of the findings to other parts of the country. Regional variations in agroecology, infrastructure, and institutional support could lead to different outcomes elsewhere. Therefore, future research should expand to multiple zones or regions to provide a broader and more representative picture of the adoption and impact of cluster farming across Ethiopia. Third, in our study, we used a dummy variable to indicate whether farmers had contact with extension services, mainly because more detailed data were not available. Although this method is common in the literature, it may introduce some bias, especially since today most farmers are assumed to have at least some access to extension support. In the future, it would be more informative to track how often farmers are visited during the cropping season, as this would provide a clearer picture of the support they actually receive. Finally, while this research provides valuable insights at the household level, future studies should explore cluster‐level dynamics to gain a deeper understanding of collective decision‐making, coordination challenges, and the broader socioeconomic and institutional factors shaping the success of cluster farming in Ethiopia.

## Conflicts of Interest

The authors declare no conflicts of interest.

## Funding

This study was funded by the Debre Berhan University.

## Endnotes

^1^For year 2023/2024, the national average exchange rate for Ethiopia, accessed from National Bank of Ethiopia quarter report, was $1 = birr 55.96.

## Data Availability

The data that support the findings of this study is available upon reasonable request from the corresponding author.

## Appendix A

**Table A1:** Falsification test for per capita consumption equation.

**PCE**	**Coef.**	**St. err.**	**t** **-value**	**p** **value**	**95% conf**	**Interval**	**Sig**
Information cluster	−1975.807	1641.936	−1.20	0.231	−5221.217	1269.603	
Perceived impact	−148.049	1279.912	−0.12	0.908	−2677.892	2381.794	
Training	−2817.954	1492.782	−1.89	0.061	−5768.549	132.641	^∗^
Distance farm	166.741	762.754	0.22	0.827	−1340.9	1674.382	
Constant	18247.297	2575.569	7.08	0	13156.49	23338.103	^∗∗∗^

Mean dependent var	14291.347		SD dependent var			6408.558	
*R*‐squared	0.034		Number of obs			149	
*F*‐test	1.256		Prob > *F*			0.290	
Akaike crit. (AIC)	3038.816		Bayesian crit. (BIC)			3053.836	

^∗∗∗^ *p* < 0.01, and  ^∗^ *p* < 0.1.

## Appendix B

**Table A2:** Falsification test for wheat output equation.

**Wheat production**	**Coef.**	**St. err.**	**t** **-value**	**p** **value**	**95% conf**	**Interval**	**Sig**
Information cluster	2.294	2.761	0.83	0.407	−3.163	7.752	
Perceived impact	−1.028	2.143	−0.48	0.632	−5.264	3.207	
Training	−0.748	2.516	−0.30	0.767	−5.721	4.225	
Constant	42.249	4.202	10.05	0	33.944	50.554	^∗∗∗^

Mean dependent var	43.965		SD dependent var			10.748	
*R*‐squared	0.017		Number of obs			149	
*F*‐test	0.836		Prob > *F*			0.476	
Akaike crit. (AIC)	1134.954		Bayesian crit. (BIC)			1146.969	

^∗∗∗^ denotes 1% level of significance.

## Appendix C

**Table A3:** Conversion factor of tropical livestock wealth (TLU).

**No.**	**Livestock type**	**TLU measurement**
1	Bee colony	0.01
2	Horses	0.8
3	Chicks	0.01
4	Chicken	0.013
5	Sheep	0.1
6	Goat	0.1
7	Camels	1.25
8	Bulls	1.2
9	Cow	1.0
10	Mule	0.8
11	Donkey	0.7
12	Steers	1.05
13	Oxen	1.0
14	Heifers	0.8
15	Calves	0.4

*Note: Source:* FAO [71].
